# *Agrobacterium*- and a single Cas9-sgRNA transcript system-mediated high efficiency gene editing in perennial ryegrass

**DOI:** 10.3389/fgeed.2022.960414

**Published:** 2022-09-06

**Authors:** Rahul Kumar, Troy Kamuda, Roshani Budhathoki, Dan Tang, Huseyin Yer, Yunde Zhao, Yi Li

**Affiliations:** ^1^ Department of Plant Science and Landscape Architecture, University of Connecticut, Storrs, CT, United States; ^2^ Section of Cell and Developmental Biology, University of California, San Diego, CA, United States

**Keywords:** perennial ryegrass, single promoter, CRISPR/Cas9, PDS, ZmUbi1, ruby, genome editing, single transcript unit

## Abstract

Genome editing technologies provide a powerful tool for genetic improvement of perennial ryegrass, an important forage and turfgrass species worldwide. The sole publication for gene editing in perennial ryegrass used gene-gun for plant transformation and a dual promoter based CRISPR/Cas9 system for editing. However, their editing efficiency was low (5.9% or only one gene-edited plant produced). To test the suitability of the maize Ubiquitin 1 (*ZmUbi1*) promoter in gene editing of perennial ryegrass, we produced *ZmUbi1* promoter:*RUBY* transgenic plants. We observed that *ZmUbi1* promoter was active in callus tissue prior to shoot regeneration, suggesting that the promoter is suitable for Cas9 and sgRNA expression in perennial ryegrass for high-efficiency production of bi-allelic mutant plants. We then used the *ZmUbi1* promoter for controlling *Cas9* and sgRNA expression in perennial ryegrass. A ribozyme cleavage target site between the *Cas9* and sgRNA sequences allowed production of functional Cas9 mRNA and sgRNA after transcription. Using *Agrobacterium* for genetic transformation, we observed a 29% efficiency for editing the PHYTOENE DESATURASE gene in perennial ryegrass. DNA sequencing analyses revealed that most *pds* plants contained bi-allelic mutations. These results demonstrate that the expression of a single Cas9 and sgRNA transcript unit controlled by the *ZmUbi1* promoter provides a highly efficient system for production of bi-allelic mutants of perennial ryegrass and should also be applicable in other related grass species.

## Introduction

Perennial ryegrass (*Lolium perenne* L.) is one of the most popular and important bunch-type cool-season turfgrass species (Jiang and Huang, 2001; Emmons and Rossi, 2015; Grinberg et al., 2016). Owing to its rapid establishment, attractiveness, and leaf appearance, it is grown in various diverse areas such as residential lawns, national parks, athletic fields, and golf course fairways. Perennial ryegrass establishes faster than other turf species, therefore, it can be used to repair damaged lawns and athletic fields (Emmons and Rossi, 2015). In addition, perennial ryegrass is commonly used on athletic fields because of its wear and tear tolerance. However, perennial ryegrass is also susceptible to drought, extreme temperature, and diseases (Emmons and Rossi, 2015).

Modern breeding techniques such as transgenic and genome editing technologies promise to be more powerful, efficient, and precise compared to conventional breeding ones (Xiong et al., 2015; Hua et al., 2019; Ahmar et al., 2020). Transgenic technology has been used successfully to improve annual and perennial crops, but it faces social and political opposition. On the other hand, CRISPR/Cas9 assisted genome editing technology can be more acceptable for the genetic improvement of crop plants (Xiong et al., 2015; Hua et al., 2019). A simple and highly efficient genome editing system for perennial ryegrass could be helpful to genetically improve many of its traits. To date, only one study of CRISPR/Cas9 mediated genome editing of perennial ryegrass has been reported (Zhang et al., 2020). The study targeted the DISRUPTED MEIOTIC cDNA1 (*DMC1*) gene using a particle bombardment mediated-transformation method. However, only a single genome-edited perennial ryegrass plant (or 5.9% genome editing efficiency) was produced in their experiments.

Successful genome editing requires coordinated spatio-temporal expression of Cas9 protein and sgRNA, which has been achieved either by using a compatible set of two promoters or a single transcript unit (STU) system (Osakabe and Osakabe, 2015; Tang et al., 2016; Tang et al., 2019; Zhuo et al., 2021). Sometimes it can be difficult to achieve coordinated expression of Cas9 and sgRNAs in a dual promoter-based system, especially in non-model organisms where promoters have not been well characterized. A STU for CRISPR/Cas9 system relies on the expressions of Cas9 and sgRNAs under a single promoter, eliminating the need for multiple promoters working in concert. A STU CRISPR/Cas9 system has been reported in rice for high genome editing efficiencies (Tang et al., 2016). In their STU CRISPR/Cas9 system, Tang et al. (2016) co-expressed Cas9, sgRNA, and a self-cleaving hammerhead ribozyme (RZ) with a single maize Ubiquitin 1 (*ZmUbi1*) promoter. The single sgRNA and Cas9 transcripts are cleaved by the cis-acting ribozyme to generate functional Cas9 and sgRNAs.

In this study, we report the spatio-temporal activities of the *ZmUbi1* promoter in perennial ryegrass using a *RUBY* reporter construct. We further report the use of *Agrobacterium* to deliver a single *ZmUbi1* promoter based CRISPR/Cas9-sgRNA system into perennial ryegrass. Using our methodology, we have observed 29% editing efficiency in perennial ryegrass when the PHYTOENE DESATURASE (*PDS*) gene was used as a target.

## Materials and methods

### *PDS* gene sequence analysis and vector information

**FIGURE 1:**
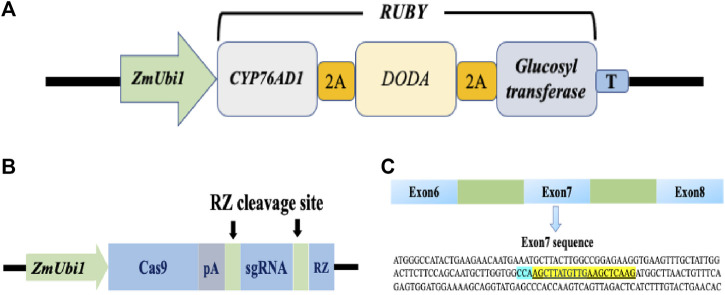
RUBY and CRISPR/Cas9 vector maps and target sequence for the perennial ryegrass PDS gene. **(A)** Schematic map of the RUBY construct. RUBY construct was designed by (He ct al., 2020). The three betalai biosynthetic genes were fused into a single open reading frame, The single transcript was expressed using a single ZmUbil promoter. 2A peptide coding sequences were inserted between these genes. A fler translation, the 2A peptides undergo self-cleavage. Thus releasing the individual proteins for betalain biosynthesss. **(B)** The ZmUbil promoter was used to contro] the expression of Cas9, PRNA, and RZ in a CRISPR/Cas9 construct. CRISPR'Cas9 construct was designed by Tang et al. (2016). A synthetic polyA (pA) sequence was used to terminate Cas9 mRNA translation. The RZ sequence (in blue) and its recognition sequence (in green) are used for producing functional Cas9 and sgRNA after transcription. **(C)** The gRNA sequence for the perennial ryegrass PDS gene.

Homologous nucleotide sequences of *PDS* gene of wheat, rice, bermudagrass, and rigid ryegrass were retrieved by using the BLAST function from the NCBI database (www.ebi.ac.uk/Tools/sss/ncbiblast/nucleotide.html). The retrieved sequences were then used for BLAST analysis in perennial ryegrass transcriptome to identify a *PDS* gene (Byrne et al., 2015). *PDS* CRISPR/Cas9 (www.addgene.org/89269/) and visual marker *RUBY* (www.addgene.org/160909/) constructs were purchased from addgene (www.addgene.org). The *RUBY* reporter contains three genes CYP76AD1, L-DOPA 4,5-dioxygenase (*DODA*), and glucosyltransferase (Figure 1A). These three genes were linked by sequences that encode self-cleaving 2A peptides, which produce three functional proteins when the 2A peptides cleave themselves after translation (He et al., 2020). The *PDS* CRISPR/Cas9 construct used in this study was previously used for genome editing in rice (Tang et al., 2016). Nucleotide sequences of perennial ryegrass and rice *PDS* gene were perfectly matched at target and PAM location. In the CRISPR/Cas9 vector, a *ZmUbi1* promoter was used to control the expression of Cas9 and gRNA (*ZmUbi1*:Cas9:gRNA). The sgRNA was flanked by RZ cleavage sites (Figure 1B). Both constructs contained a hygromycin B resistant gene as a selectable marker.

### *Agrobacterium-*mediated transformation of perennial ryegrass

Perennial ryegrass (*L. perenne L.*) cultivar “Fiesta-4” seeds were used for embryogenic callus induction. The seeds were de-husked by soaking them in 50% H_2_SO_4_ for 30 min. The de-husked seeds were surface sterilized with 3% (w/v) sodium hypochloride for 15 min, then washed several times with sterile distilled water. Sterilized seeds were bisected longitudinally with a sterilized blade into two parts, then cultured on a callus induction medium (3.98 g/L N6 basal salts, 1 mg/L thiamine-HCL, 1 g/L Casein hydrolysate, 800 mg/L proline, 20 mg/L L-Gln, 10 mg/L lipoic acid, 5 mg/L 2,4-D, 30 g/L maltose, 0.1 mg/L 6-benzyladenine (BA), and 3 g/L phytagel, pH 5.9). After the first week, growing shoots were cut at 4–6 days intervals and the callus induction medium was changed every 3 weeks. The CRISPR/Cas9 and *RUBY* vectors DNA were transferred into the *Agrobacterium tumefaciens* strain EHA105 (Wise et al., 2006) and transferred into embryogenic calli of perennial ryegrass using an *Agrobacterium*-mediated transformation method (Patel et al., 2013). The infected calli were subsequently cultured on a co-culture medium for 3 days at 25°C in a dark growth chamber. The infected calli were transferred to a resting medium (Callus induction medium and 200 mg/L timentin, pH 5.9) at 25°C in the dark for 5 days. Infected calli were subsequently grown on a selection medium (Callus induction medium, 200 mg/L timentin, and 50 mg/L hygromycin, pH 5.9) at 25°C in a dark growth chamber for 45 days, transferring the calli to a fresh selection medium every 15 days. Hygromycin resistant calli were transferred to a shoot regeneration medium (MS salts, 0.5 mg/L 6-benzyladenine (BA), 30 g/L maltose, 3 g/L phytagel, 150 mg/L timentin, and 50 mg/L hygromycin, pH 5.9) for the regeneration at 22°C under fluorescent light (16h light/8h dark).

### Confirmation of genome editing via HindIII digestion and DNA sequencing

Transgenic plants were identified by the polymerase chain reaction (PCR) assay using a direct-PCR approach (Phire Plant Direct PCR Kit, Thermo Fisher Scientific Co., United States) according to the manufacturer’s protocol. A pair of Cas9 based primers (F- GAG​GAT​GCT​CGC​TTC​TGC​TG and R- CGA​GGT​TTG​CAT​CAG​CGA​GG) was designed to amplify a 229 bp long region to identify transgenic plants. Another pair of primers from the perennial ryegrass *GA20ox1* gene (F-TCTGACGAGAACACCCTTGA and R-AGCACCTCCA TGATCTCCAG) was designed to amplify a 94 bp long region for internal control. Another pair of primers (F-GGG​CCA​TAC​TGA​AGA​ACA​ATG and R-TTCATTTAT GGACCTAGCC ACG) flanking the target site was used to amplify the 302 bp sgRNA-target region for HindIII digestion and Sanger DNA sequencing. A 6 bp long HindIII restriction site was located adjacent to the protospacer-adjacent motif (PAM) sequence, therefore, any mutation that occurred in the restriction site will be detected by HindIII digestion. The amplified PCR products were digested by HindIII restriction enzyme (New England Biolabs), as per the manufacturer’s instructions. For the Sanger DNA sequencing, the PCR products were run in 2% agarose gel, and specific bands were eluted and purified by using Nucleospin Gel and PCR Clean-up kit (Machery-Nagel #740609). For the Illumina sequence analysis, a 199 bp sgRNA-target region was amplified using a pair of primers, and these primers (F-ACACTCTTTCCCTACACGACGCTC TTCCGATCTTGGGCCATACTGAAGAACAATG and R-G TGACTGGAGTTCAGACGTGTGCTCTTCCGATCTGAGA CG GCTATGTGTTCAGTAC) had overhanging sequences (underline) with them for sequencing. The PCR products from all *pds* mutants were sequenced using Illumina MiSeq plateform. The raw Illumina reads were mapped to the 199 bp reference perennial ryegrass *PDS* gene sequence using bwa mem (-c 300000 -v 2) of BWA v0.7.17 (Li and Durbin, 2009; Chen et al., 2018).

## Results

### *ZmUbi1* promoter shows activity in transformed calli and regenerating shoots

**FIGURE 2:**
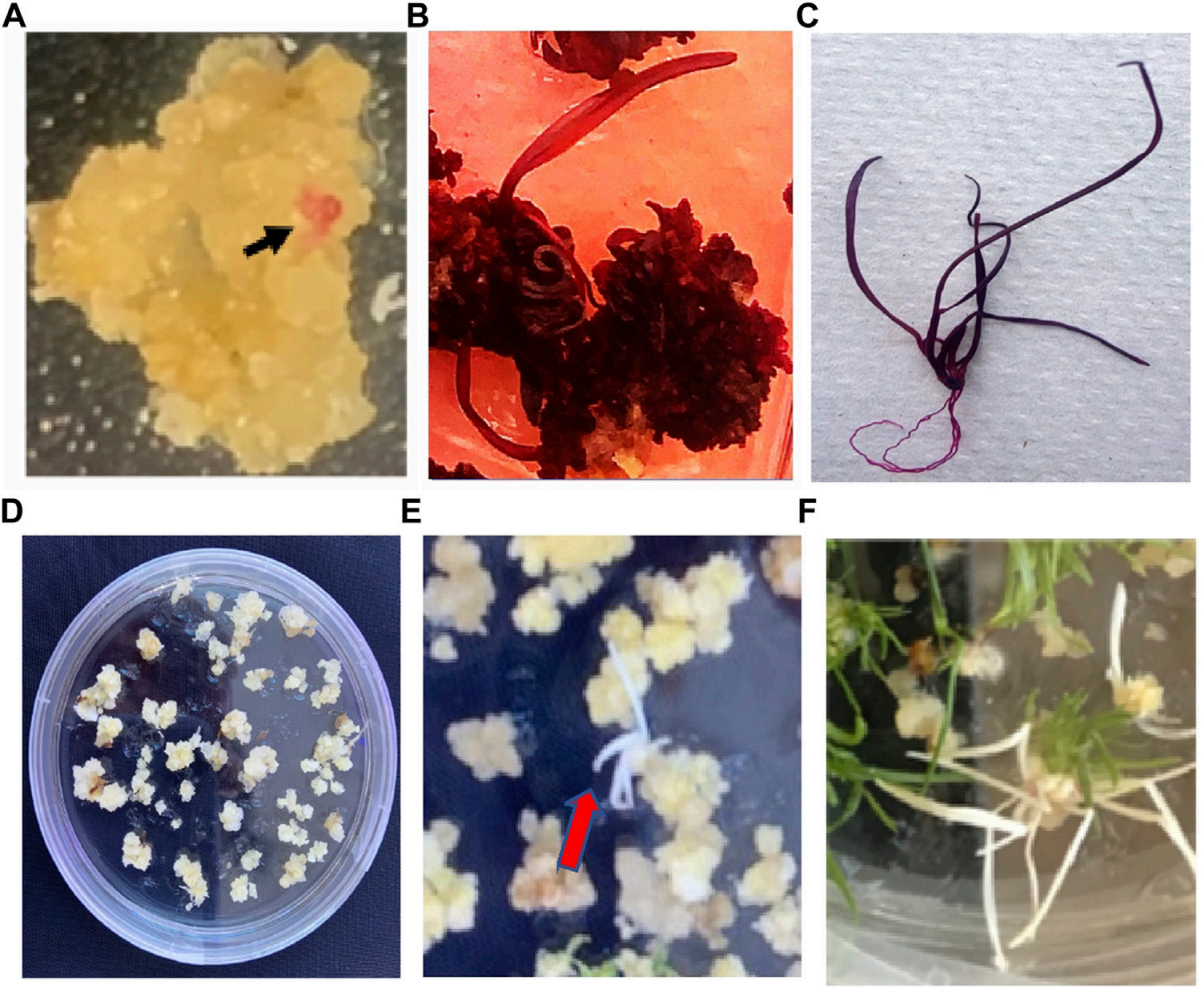
The activity of the ZmUbil promoter driving RUBY and production of pds mutants of perennial ryegrass. The ZmUbsl promoter:RUBY gene was expressed in call **(A)** (indicated by black arrows), shoots **(B)**, and root and shoot **(C)**. Putative CRISPR'Cas9 transformed perennial ryegrass calius under 50 mg/L hygromycin selection **(D)**. Regenerating PDS genc-edited albino plants at the carty stage as indicated by orange arrows **(E)**. PDS gene-cdited perennial ryegrass plant show mg albino phenotype **(F)**.

*RUBY* is a novel reporter gene system for higher plant that produces a red color pigment, betalain, if expressed (He et al., 2020). We investigated the spatio-temporal activities of the *ZmUbi1* promoter in perennial ryegrass using the easily visible *RUBY* reporter. The *RUBY* construct was delivered in perennial ryegrass calli via an *Agrobacterium*-mediated transformation method. The use of *RUBY* made it easier to distinguish the transformed cells from untransformed ones owing to the red coloration. We observed that red color in explants 3 days post the *Agrobacterium* infection, suggesting that the *ZmUbi1* promoter is active at a very early stage of transformation (data not shown). We produced twenty-three independents transgenic *RUBY* calli and 22 transgenic independent *RUBY* plant lines. As shown in Figures 2A–C–C, the *ZmUbi1* promoter is active in calli and throughout the plant life cycle in roots, shoots and leaves. Because the *ZmUbi1* promoter is active in calli before shoot regeneration, it should be a good candidate for controlling the expression of Cas9 and sgRNAs in perennial ryegrass to allow genome editing at the single-cell (callus) stage.

### Elevated hygromycin concentrations reduce natural albinism in perennial ryegrass generated from tissue culture

Spontaneous albinism is a common problem encountered during tissue culture and regeneration of perennial ryegrass without genetic transformation (Liu et al., 2006). The presence of these naturally occurring albino plants could complicate the identification of gene-edited *pds* mutant plants. To reduce that problem, we tested the effects of concentrations of hygromycin in the transgenic cell/plant selection medium on albinism frequency. A construct with no Cas9 or sgRNA with hygromycin resistance was used as a control when examining the effects of hygromycin on spontaneous albinism. As shown in Table 1, 16% of regenerated plants in regeneration medium without hygromycin were found to be albino. These albino plants were able to survive on culture media for months. When hygromycin was used in the media, we observed reductions in natural albinism (Table 1). Addition of 10, 20, and 30 mg/L hygromycin in the media resulted in production of 13, 10, and 4% albino plants, respectively. A complete suppression of natural albino plants was observed with 50 mg/L hygromycin in the media. Wild type perennial ryegrass albino shoots were not capable of tolerating 50 mg/L hygromycin, which led to the elimination of spontaneous albinism and also allowed more effective selection of transgenic plants and *pds* mutant plants.

**TABLE 1:** Effects of hygromycin concentrations on natural albino plant percentage in perennial ryegrass tissue culture, transformed with a control vector containing no Cas9. Hygromycin resistant calli were transferred to a shoot regeneration medium supplemented with 0, 10, 20, 30, and 50 mg/L hygromycin.

Hygromycin concentration (mg/L)	Total plants	Total albino plants	Albino plants (%)
0	605	99	16.1
10	303	40	13.2
20	235	24	10.2
30	270	10	3.7
50	340	0	0

### Production of *pds* albino perennial ryegrass plants by targeted mutagenesis

The two sgRNAs in the CRISPR/Cas9 construct used in this study was originally designed for the rice *PDS* gene (Tang et al., 2016). One of these two sgRNAs is perfectly matched with the perennial ryegrass *PDS* gene sequence but another rice sgRNA is not (Figure 1S). Therefore, the characterization of mutations in the edited *pds* mutants of perennial ryegrass was on the sequence well matched with the rice sgRNA sequence. The CRISPR/Cas9 and control (no Cas9/sgRNA) constructs containing the hygromycin phosphotransferase gene were delivered in calli via an *Agrobacterium*-mediated transformation method. The embryogenic calli were subsequently transferred into a selection medium containing hygromycin (50 mg/L) consecutively for 45 days (Figure 2D). Hygromycin-resistant calli were transferred to a shoot regeneration medium containing hygromycin (50 mg/L). Shoots were visible 2 weeks after transferring calli to the shoot regeneration medium (Figures 2E,F). We did three transformation experiments and observed 25, 27, and 33% editing efficiency for the *PDS* gene, respectively (Table 2). Also, as expected, all albino plants from the three transformation experiments had mutations in the *PDS* gene, indicating that 50 mg/L hygromycin in shoot regeneration medium effectively eliminated natural albino plants.

**TABLE 2:** Production of *pds* perennial ryegrass mutants by targeted mutagenesis. Genome editing was confirmed by HindIII digestion and Sanger, and high-throughput Illumina sequencing.

Experiment	Total transgenic plants	Total *pds* mutants	*Pds* mutants (%)
Experiment-1	15	5	33.3
Experiment-2	12	3	25.0
Experiment-3	11	3	27.3
Total	38	11	28.9

### Confirmation of genome editing by HindIII digestion and sanger DNA sequencing

**FIGURE 3:**
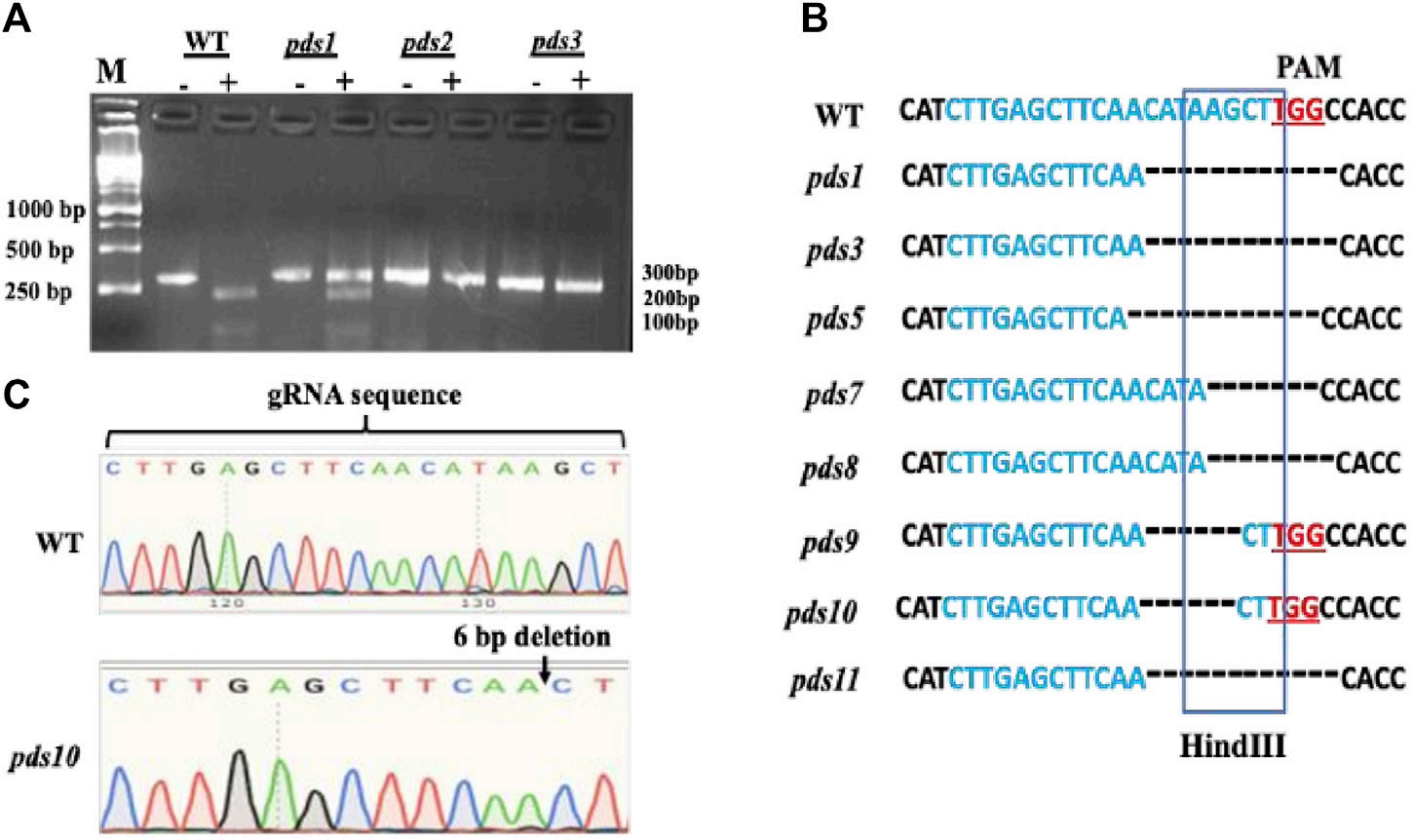
Confirmation of editing of the PDS gene by Hindlll digestion and Sanger DNA sequencing method, **(A)** PDS gene editing was confirmed by Hindfll digestion of PCR products. Hindlll treated PCR DNA products were run im an agarose gel. The “—” symbol indicates PCR product without restriction digestion and the “+” symbol indicates products after the restricoon digestion. PDS gene-edited pkints have mutations in the restriction site as aresuk their PCR products were not digested. **(B)** Mutations were confirmed by Sanger DNA sequencing of the gRNA-target region. The “—” symbol indicates muckotides deletion in the pds matants: by CRISPR/Cas9. **(C)** Sanger DNA sequencing sgRNA region of the chromosome DNA from the WT and pds10 masant depicting a 6 bp deletion in the target region.

Mutations in the *PDS* gene in the albino plants were confirmed using HindIII digestion and Sanger DNA sequencing. The Cas9 cleavage site coincides with the HindIII restriction enzyme recognition sequence; thus, the genome editing at the HindIII site led to disruption of the HindIII digestion. We used HindIII digestion to confirm mutations in the *PDS* gene in the albino plants. A pair of primers flanking the target site was used to amplify a 302 bp sgRNA-target region. A HindIII digestion of the wild type PCR product produced two bands, 214 bp and 88 bp, respectively (Figure 3A, Figure 2S). Out of 11 plants, 10 albino plants showed undigested PCR products, indicating mutations in the HindIII enzyme recognition site. Seven plants had shown completely undigested products suggesting that the *PDS* gene in these plants had been mutated completely. The presence of the HindIII digested products suggests no mutations in the *PDS* gene or mutations outside the HindIII recognition site. A 302 bp PCR amplified fragment containing the sgRNA targeting region from albino plants was sequenced using a Sanger DNA sequencing method to initially identify mutations in the albino plants. The results show various deletions in the targeted region and are consistent with the HindIII digestion results (Figures 3B,C, Figure 3S). However, we recognize that our Sanger sequencing could not identify multi-allelic mutations because we did not sub-clone PCR products into an *E. coli* vector and sequence these clones.

### Illumina sequencing of targeted mutations in perennial ryegrass *pds* mutants

To reveal multi-allelic mutations in the *pds* mutants, we used Illumina sequencing for a more detailed characterization of the mutations in representative *pds* mutant plant. A 199 bp long DNA spanning the targeted region was amplified from five representative albino plants and used for Illumina sequencing analysis. The sequencing results of the target region were shown in Table 3. The selected *pds* mutant plants had a variety of mutated sequences at and near the PAM sequence of the target site. Mutations were identified in all five plants with 1–12 bp deletions in the target region. Four plants had bi-allelic mutations, suggesting that genome editing mainly took place at the single-cell stage of shoot regeneration. Furthermore, a majority of mutants contained 1 to 2 bp deletions which result in a frame shift of the coding sequence. In addition, the sequencing results also showed that *pds*4 mutant contained bi-allelic mutations outside of the HindIII restriction site which could not be detected by digestion. However, we did not observe any insertion events in these mutant plants. The Illumina sequencing analysis show that most *pds* mutant plants we produced contained bi-allelic mutations, which reduces chances for production of chimeric plants.

**TABLE 3:** Illumina sequencing results of targeted mutations in *pds* mutants.

Plant	Allele	Mutation	Indel type	Sequence (%)
WT	WT sequence	CTT​GAG​CTT​CAA​CAT​AAG​CTT​GGC​CAC​C	-	100
*pds1*	Allele 1 (WT)	CTT​GAG​CTT​CAA​CAT​AAG​CTT​GGC​CAC​C	-	28
	Allele 2	CTT​GAG​CTT​CAA________________CAC​C	-12	34
	Allele 3	CTT​GAG​CTT​CAA​CA_AAG​CTT​GGC​CAC​C	-1	12
	Allele 4	CTT​GAG​CTC​CAA​CAT​A_GCT​TGG​CCA​CC	-1	26
*pds2*	Allele 1	CTT​GAG​CTT​CAA​CAT__GCT​TGG​CCA​CC	-2	50
	Allele 2	CTT​GAG​CTT​CAA​CA_AAG​CTT​GGC​CAC​C	-1	50
*pds3*	Allele 1	CTT​GAG​CTT​CAA________________CAC​C	- 12	50
	Allele 2	CTT​GAG​CTT​CAA​CAT__GCT​TGG​CCA​CC	-2	50
*pds4*	Allele 1	CTT​GAG​CTT​CAA​C_TAA​GCT​TGG​CCA​CC	-1	50
	Allele 2	CTT​GAG​CTT​CAA__TAA​GCT​TGG​CCA​CC	-2	50
*pds6*	Allele 1	CTT​GAG​CTT​CAA​CAT__GCT​TGG​CCA​CC	-2	50
	Allele 2	CTT​GAG​CTT​CAA​CAT​A_GCT​TGG​CCA​CC	-1	50

## Discussion

CRISPR/Cas9 systems have become powerful tools for targeted mutagenesis to study gene functions and improve crop plants. However, an efficient CRISPR/Cas9 genome editing system in perennial ryegrass is needed. In the current study, we used a single promoter based CRISPR/Cas9 system for targeted mutagenesis in perennial ryegrass using an *Agrobacterium*-mediated transformation method. Using *ZmUbi1* promoter:*RUBY* transgenic plants, we demonstrated that *ZmUbi1* promoter was active in callus tissues, young shoots, and entire plants, suggesting that the promoter is a good candidate for driving Cas9 and sgRNA expression in perennial ryegrass. Combining *Agrobacterium*-mediated transformation with a single promoter based CRISPR/Cas9 system led to a 29% gene editing efficiency in perennial ryegrass, which is a drastic improvement from the previously published protocol (Zhang et al., 2020). Further, most of the mutants produced using our system contain bi-allelic mutations.

The promoter used to drive the expression of Cas9 and sgRNAs is a key component influencing the efficiency of gene editing and also the likelihood of bi-allelic, or multi-allelic mutants in diploid plants (Yan et al., 2015; Zhang et al., 2017). If tissue culture/plant regeneration procedures are used for development of gene edited plants, promoters that are active in callus cells before differentiated into shoots or embryos should be good choices for high efficiencies of gene-editing and bi-allelic mutant plant production (Ma et al., 2015; Wang et al., 2015; Osakabe et al., 2016; Zhuo et al., 2021). Because few promoters have been characterized in perennial ryegrass and other turf grasses, our evaluation of the spatial and temporal expression patterns of the *ZmUbi1* promoter in perennial ryegrass generates useful information for the utility of the promoter in gene editing and other applications. That fact that most of our *pds* mutant plants we produced have bi-allelic mutations indicates that gene editing took place during the single-cell stage of shoot regeneration, which further confirms the *ZmUbi1* promoter is a good choice for controlling Cas9 and sgRNA expression. Also, the high activity the *ZmUbi1* promoter in callus cells should be a basis for the relatively high gene editing efficiency we have observed.

The use of the STU (single transcription unit) system for both Cas9 and sgRNA helps to achieve a coordinated expression of Cas9 and sgRNAs in the same cell at the same time, which should also contribute to high editing efficiency reported here. Coordinated expression of Cas9 and sgRNAs can sometimes be a challenge if two different gene promoters are used to control the expression of Cas9 and sgRNA, respectively (Tang et al., 2019). The sole perennial ryegrass gene editing study published was done by using a dual promoter based CRISPR system (Zhang et al., 2020). In addition to the fact that they produced only one gene edited plants, the editing in that edited plant was about 75% (Zhang et al., 2020). While we conclude that STU contributes to higher editing efficiency as we reported here, it is also possible sgRNA sequences used may contribute to the differences in the editing efficiencies between a STU we used and a two transcript system reported by Zhang et al. (2020). On the other hand, with the STU CRISPR/Cas9 system to co-express Cas9 and sgRNA, along with the use of a hammerhead ribozyme system (Tang et al., 2016; Tang et al., 2019), functional Cas9 mRNA and sgRNAs can be produced from a single transcript. Such an expression system leads to expression of Cas9 and sgRNAs in the same cell at the same time, which should contribute to the high editing efficiencies in perennial ryegrass as we have observed. We therefore suggest that a STU CRISPR/Cas9 system should be a better choice for coordinated expression of both Cas9 and sgRNAs in order to achieve high editing efficiencies for perennial ryegrass and other closely related species.

*Agrobacterium* and particle bombardment transformation methods have been used for genetic transformation of perennial ryegrass, but *Agrobacterium* is a much simpler and easier method with high efficiency (Hansen and Wright, 1999; Travella et al., 2005; Zhang and Zheng, 2008; Patel et al., 2013; Zhang et al., 2013; Zhang et al., 2020). Zhang et al. (2020) used a particle bombardment-mediated transformation method in their CRISPR/Cas9-mediated gene editing study, but their transformation efficiency was 2.66%. In our case, we used *Agrobacterium*-mediated transformation to deliver the Cas9 and sgRNA into perennial ryegrass and our transformation efficiency was 20%, consistent with the transformation efficiencies previously reported (Patel et al., 2013; Zhang et al., 2013). Thus, we suggest that *Agrobacterium* should be used for delivery of Cas9 and sgRNAs into perennial ryegrass for high editing efficiency.

In summary, we have demonstrated a significant improvement in genome editing efficiency in perennial ryegrass compared to the only published method (Zhang et al., 2020). The key component of our method is to use *Agrobacterium* to deliver the CRISPR/Cas9 system that produces a STU for Cas9 and sgRNA driven by the *ZmUbi1* gene promoter. The method described here should also be applicable for other related grass species.

## Data Availability

The datasets presented in this study can be found in online repositories. The names of the repository/repositories and accession number(s) can be found below: https://www.ncbi.nlm.nih.gov/, SAMN26236011; https://www.ncbi.nlm.nih.gov/, OM849229-OM849237.
