# Supplementary material for: Agrobacterium- and a single Cas9-sgRNA transcript system-mediated high efficiency gene editing in perennial ryegrass
Source: Front Genome Ed. 2022 Sep 6;4:960414. doi: 10.3389/fgeed.2022.960414 (PMC9485938; doi:10.3389/fgeed.2022.960414)
Supplement: Supplementary file 1 [file DataSheet1.docx]

**Target PAM**

**A**

*LpPDS* CCTGCTTTTCCATCCACTCTGAAACAGTTAAGCCATCTTGAGCTTCAACATAAGCTTGGC

O*sPDS*  CCTGCTTTTCCATCCACTCTGAAACAGTTAAGCCATCTTGAGCTTCAACATAAGCTTGGC

************************************************************

*LpPDS* CACCAAGCATTGCTGGAAGAAGTCCAATAGCAAACTTCACCTTCTCCGGCCAAGTAAGCA

*OsPDS*  CACCAAGCATTGCTGGAAGAAGTCCAATAGCGAACTTCACCTTCTCCGGCCAAGTAAGCA

******************************* ****************************

*LpPDS*  TTTCATTGTTCTTCAGTATGGCCCATATTCCTGTTAAA

*OsPDS*  TTTCATTGTTCTTCAGTATGGCCCATATTCCGTTTAAA

******************************* *****

**Mismatch target PAM**

**B**

*LpPDS*  AGGAGTATTATGATCCAGACCGTTCAATGCTGGAGTTGGTGTTTGCTCCAGCAGAGGAAT

*OsPDS*  AGGAATACTATGATCCAAGCCGTTCAATGCTGGAGTTGGTCTTTGCTCCTGCAGAGGAAT

**** ** ********* ********************* ******** **********

*LpPDS* GGATTGGACGTAGCGACGCTGAAATCATCGAAGCAACCATGCAAGAGCTAGCCAAGTTAT

*OsPDS* GGGTTGGACGGAGTGACACTGAAATCATCGAAGCAACTATGCAAGAGCTAGCCAAGCTAT

** ******* ** *** ******************* ****************** ***

*LpPDS*  TTCCTGATGAAATAGCTGCTGATCAGAGTAAAGCAAAAATTCGTAAATACCATGTTGTGA

*OsPDS*  TTCCTGATGAAATTGCTGCTGATCAGAGTAAAGCAAAGATTCTGAAGTATCATGTTGTGA

************* *********************** **** ** ** **********

*LpPDS* AGACGCCGAG

*OsPDS*  AGACACCAAG

**** ** **

**Supplementary figure S1.** **Nucleotide similarity of PDS gene in perennial ryegrass (*LpPDS*) and rice (*OsPDS*).** Nucleotide sequence of perennial ryegrass *PDS* gene was blasted with rice *PDS* gene (AF049356.1) using online clustalw tool ([www.genome.jp/tools-bin/clustalw](http://www.genome.jp/tools-bin/clustalw)). **(A)** CRISPR/Cas9 construct designed to target rice *PDS* gene had 100% similarity with perennial ryegrass *PDS* gene (exon 7) at the target and PAM locations. **(B)** At the Second target, perennial ryegrass *PDS* gene (exon 13) had two mismatch with rice *PDS* gene and gRNA.

**
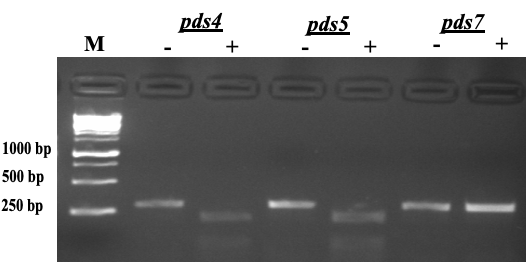

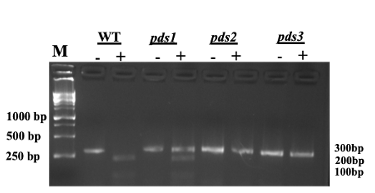
**

**
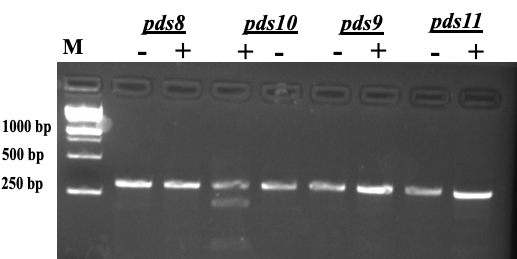
**

**Supplementary figure S2. Confirmation of editing of the *PDS* gene by HindIII digestion.** *PDS* gene editing was confirmed by HindIII digestion of PCR products. HindIII treated PCR DNA products were run in an agarose gel. The “–” symbol indicates PCR product without restriction digestion and the “+” symbol indicates products after the restriction digestion. *PDS* gene-edited plants have mutations in the restriction site as a result their PCR products were not digested.

***pds1***


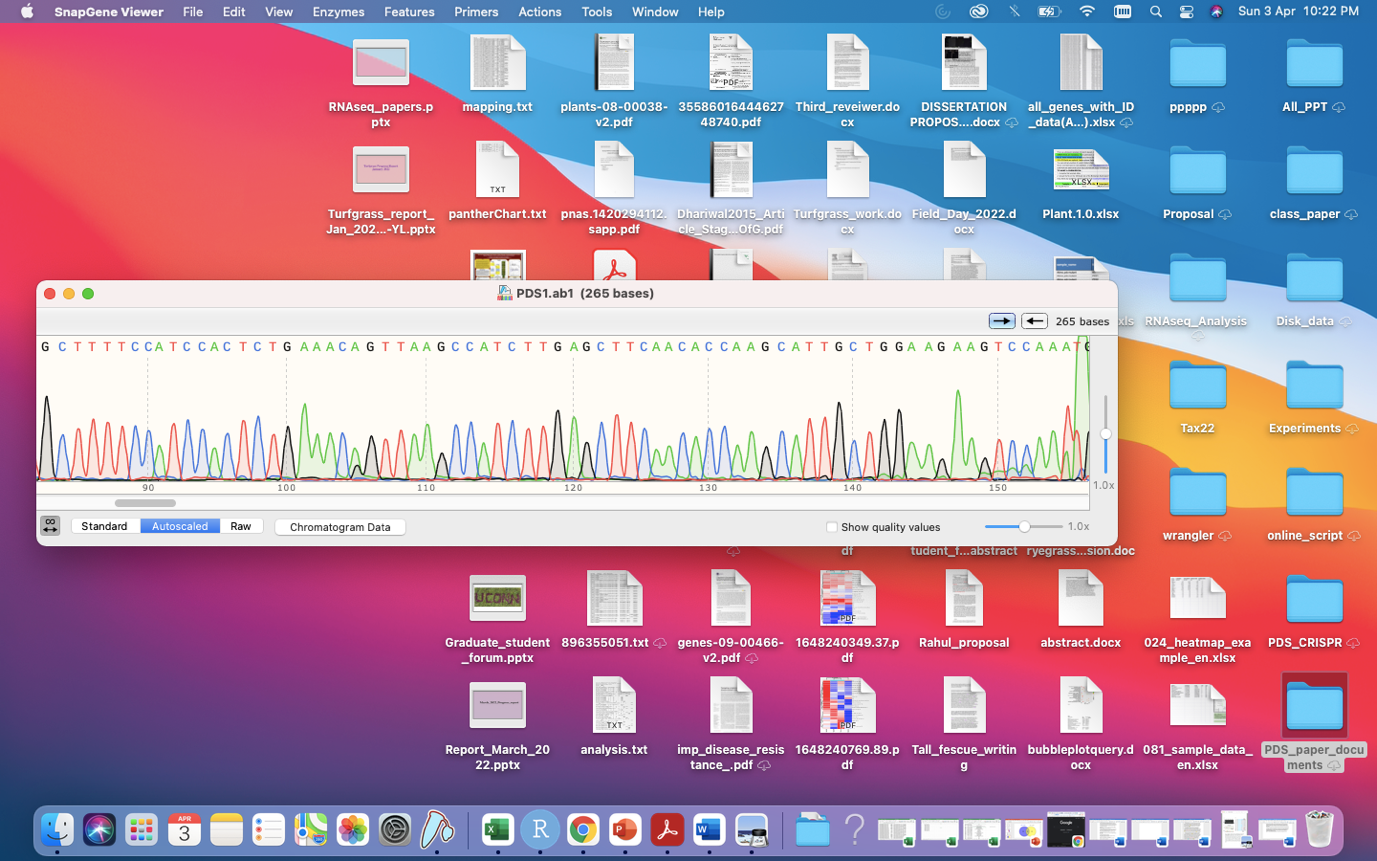


***pds3***


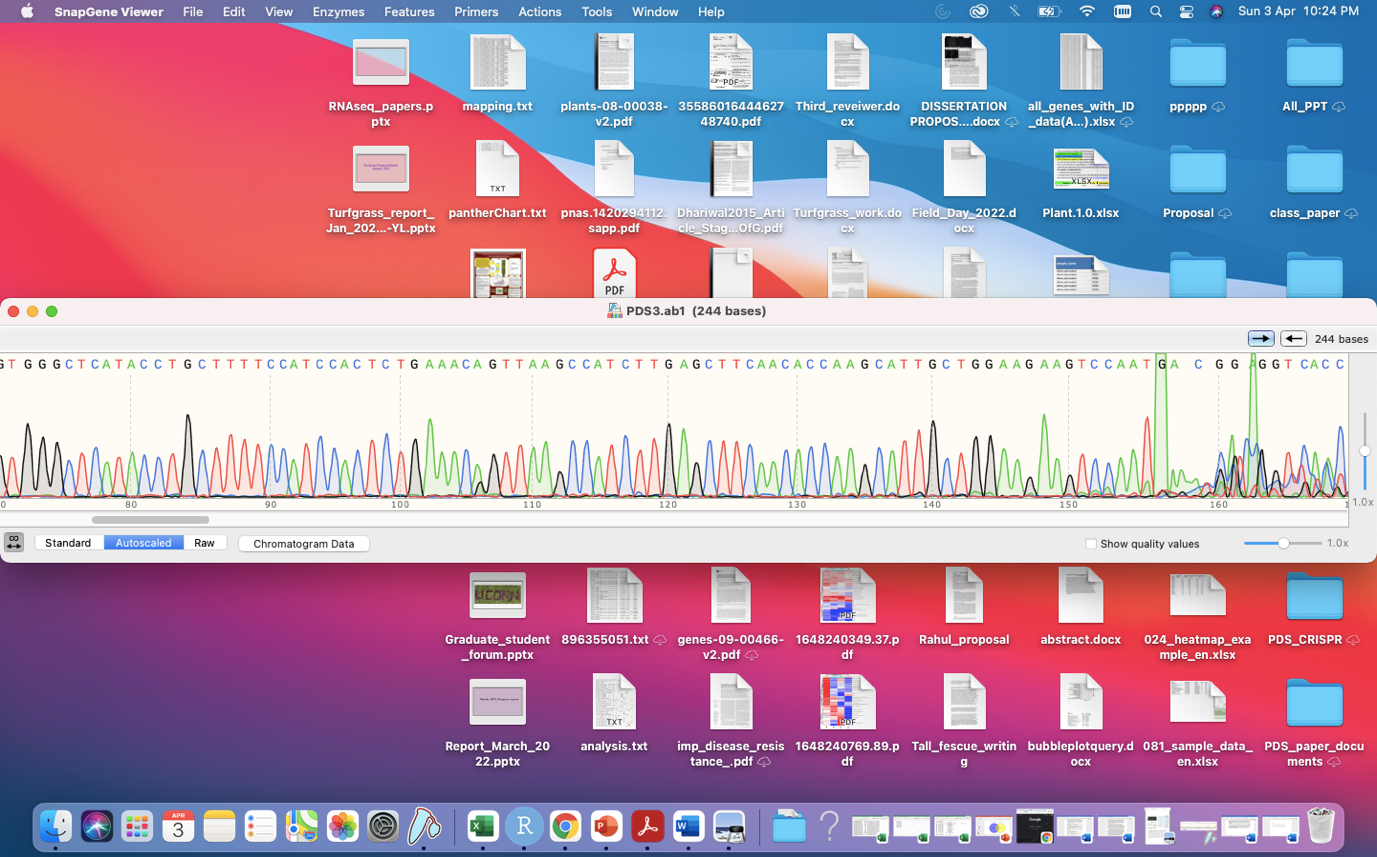


***pds5***


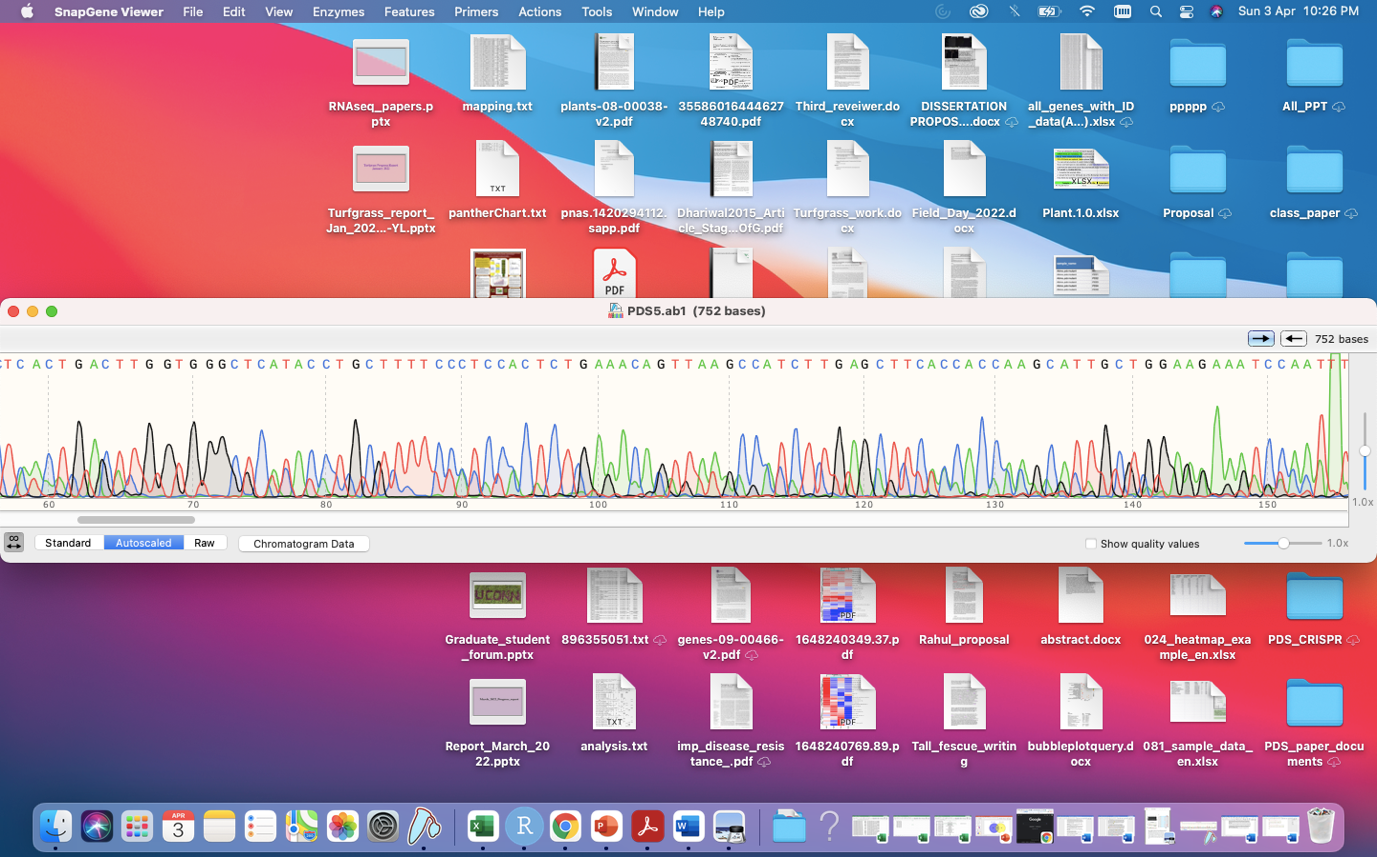


***pds7***


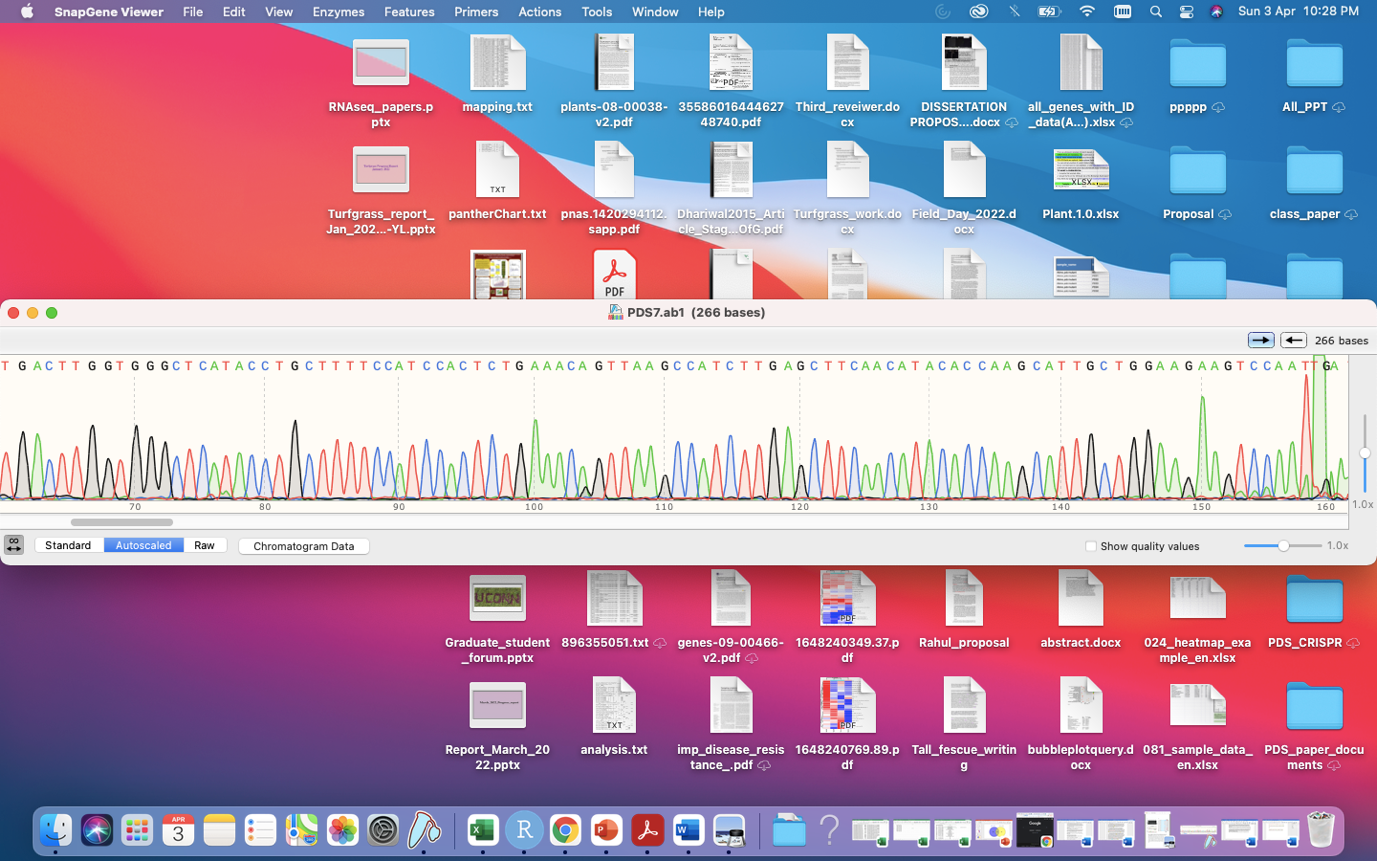


***pds8***


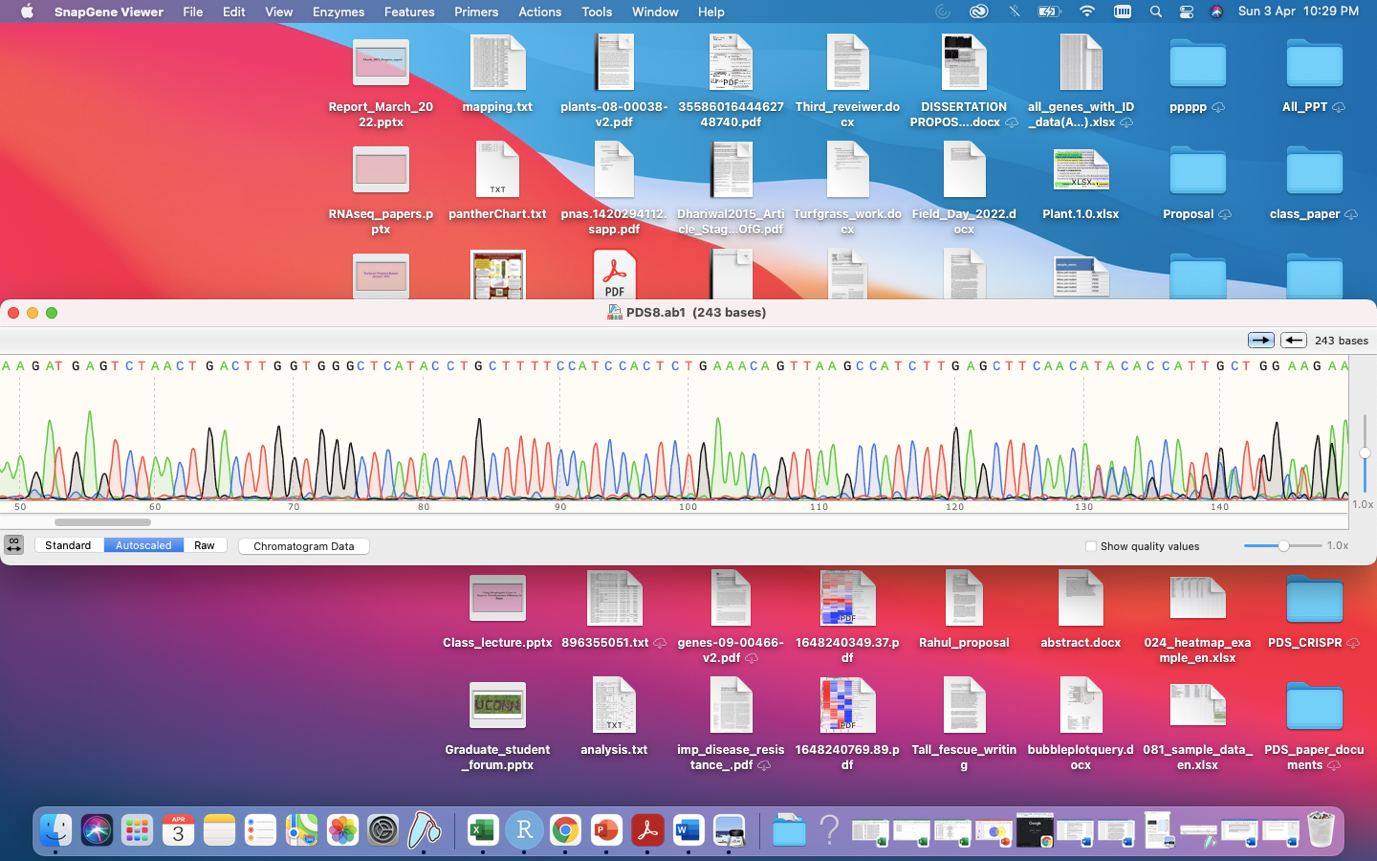


***pds9***


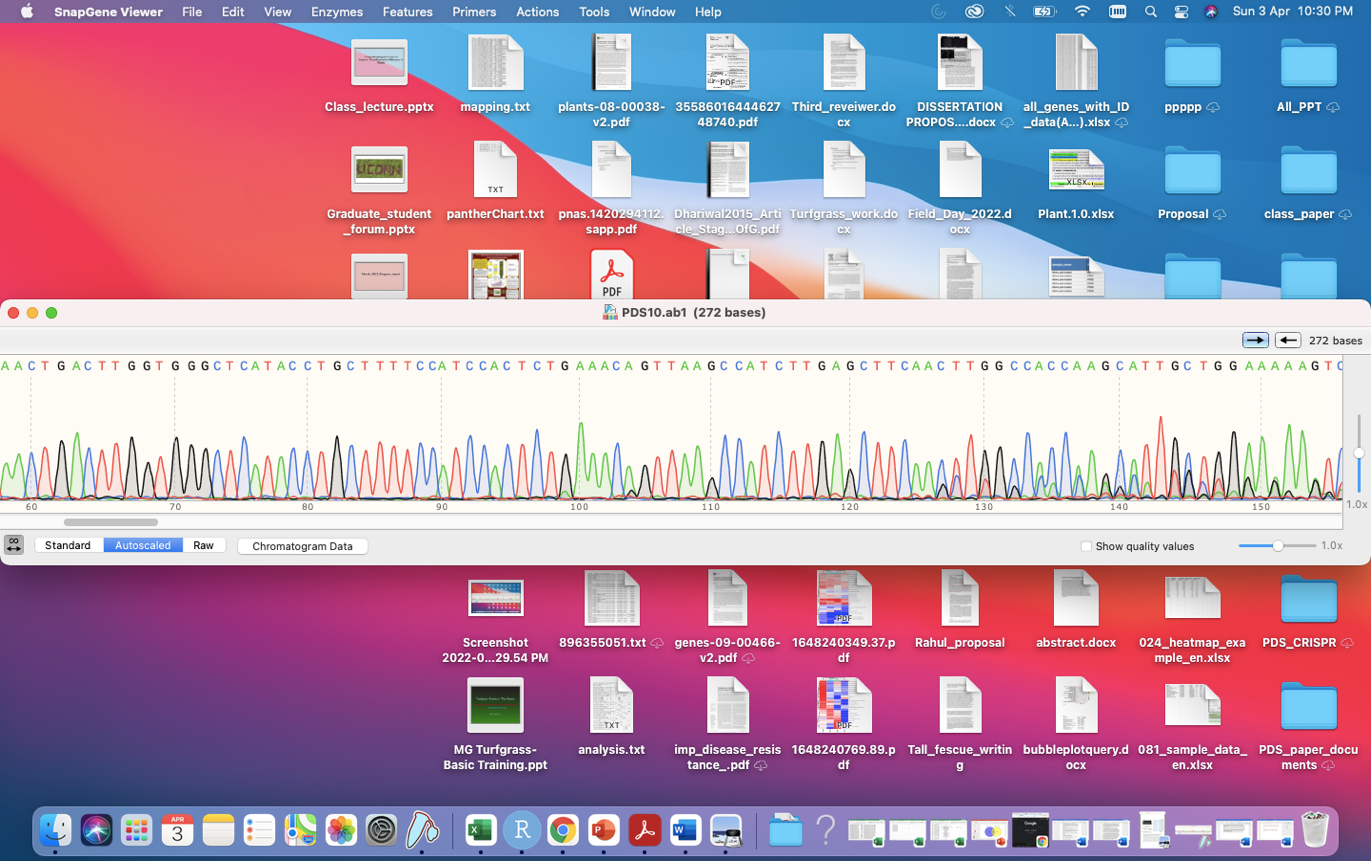


***pds10***


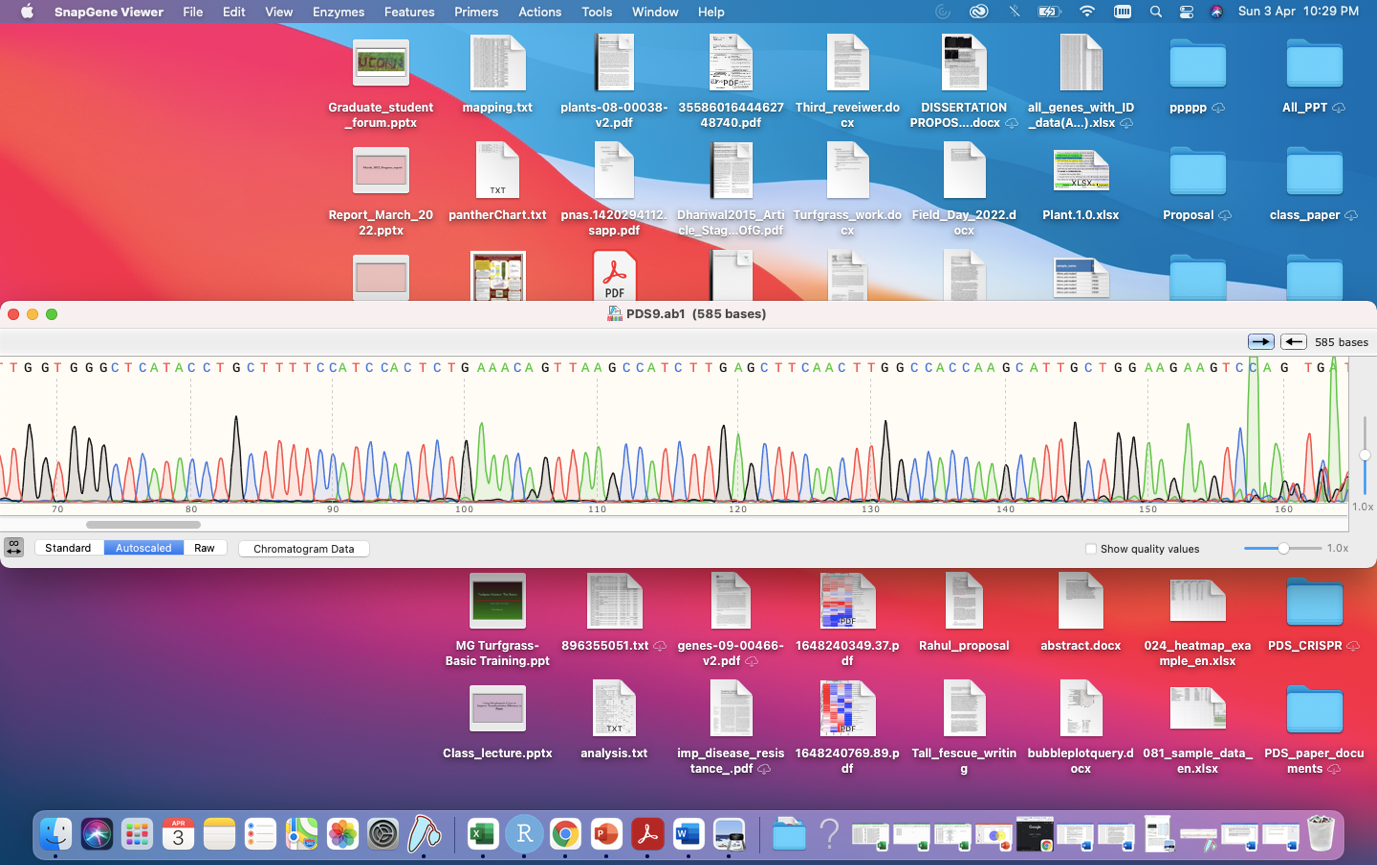


***pds11***


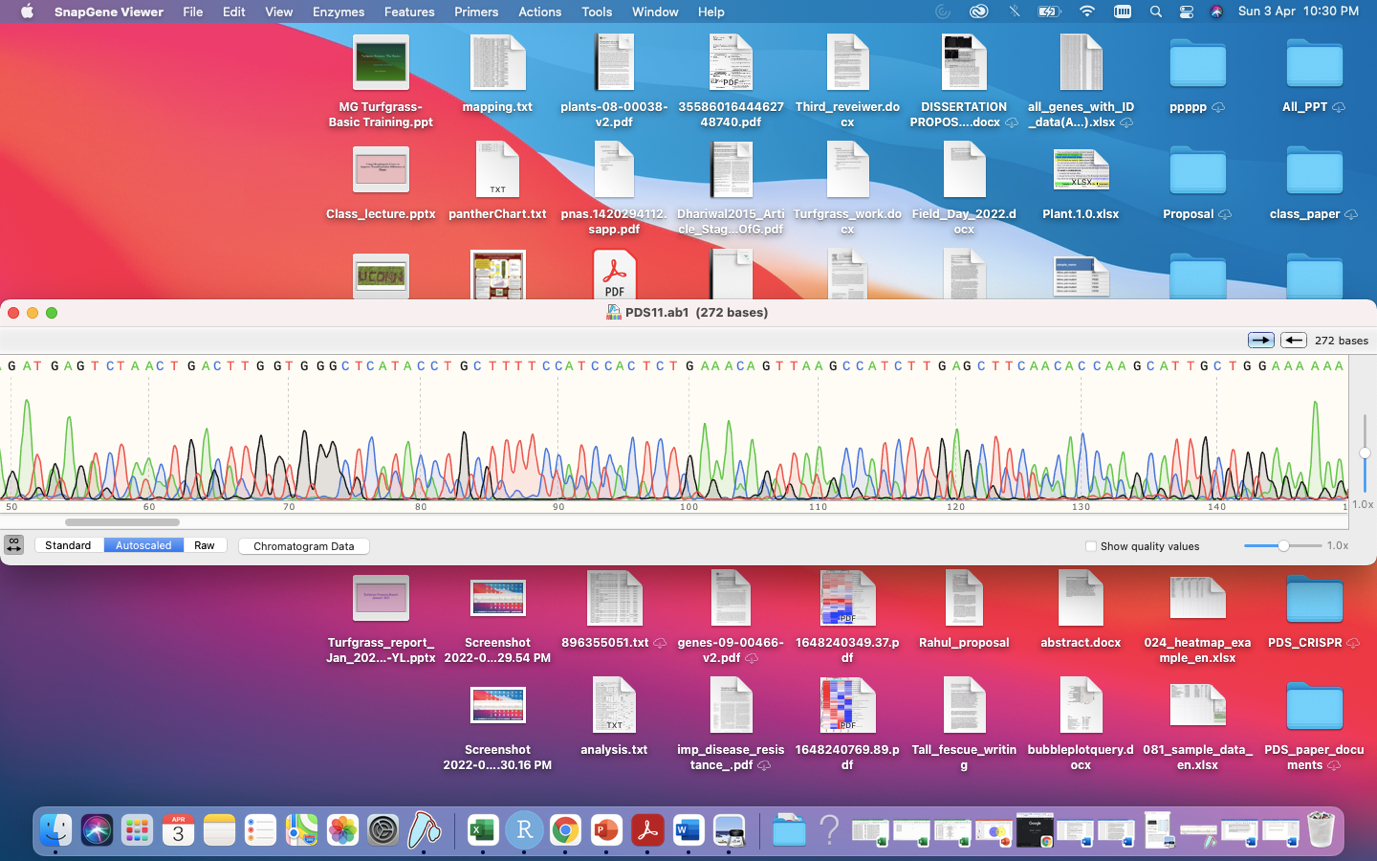


WT


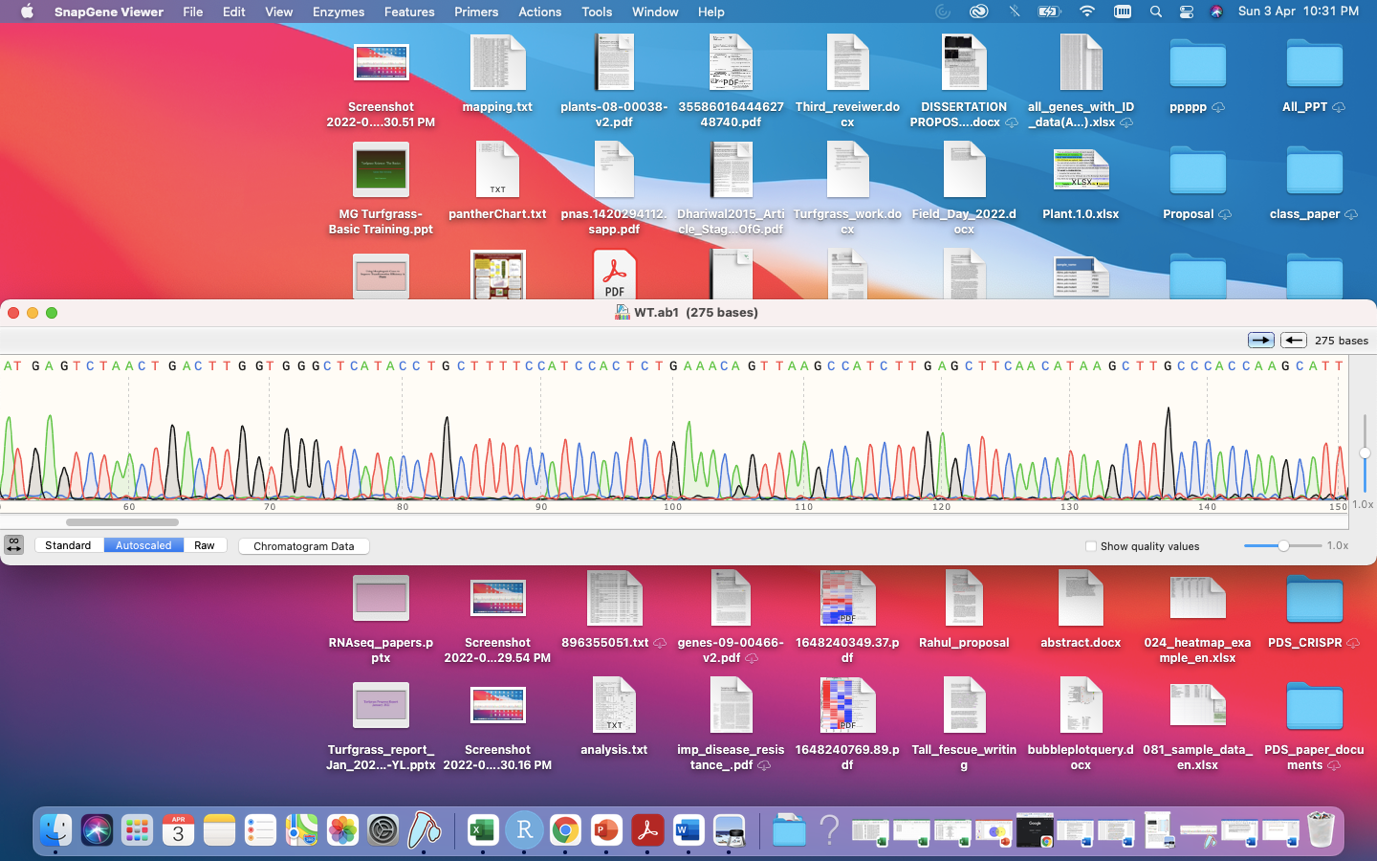


**Supplementary figure S3. Sanger sequencing chromatograms of the *pds* mutants.** PCR products of *pds1, pds3*, and *pds7* mutants were cloned into *E. coli* vector for Sanger sequencing. PCR products of *pds5, pds8*, *pds9*, *pds10*, and *pds11* mutants were sequenced directly from PCR products.
